# Real-world analysis of different intracranial radiation therapies in non-small cell lung cancer patients with 1–4 brain metastases

**DOI:** 10.1186/s12885-022-10083-8

**Published:** 2022-09-24

**Authors:** Zhengting Chen, Lingli Zhou, Min Zhao, Ke Cao, Yanqing Li, Xiaoling Liu, Yu Hou, Lan Li, Li Wang, Li Chang, Mei Yang, Wenhui Li, Yaoxiong Xia

**Affiliations:** 1 Department of Radiation Oncology, The Third Affiliated Hospital of Kunming Medical University (Yunnan Cancer Hospital, Yunnan Cancer Center), Kunming, 650118 Yunnan China; 2 Department of Medical Administration, The Third Affiliated Hospital of Kunming Medical University (Yunnan Cancer Hospital, Yunnan Cancer Center), Kunming, 650118 Yunnan China; 3 Department of Cadre Medical, The Third Affiliated Hospital of Kunming Medical University (Yunnan Cancer Hospital, Yunnan Cancer Center), Kunming, 650118 Yunnan China

**Keywords:** Stereotactic radiosurgery, Brain metastases, Non-small cell lung cancer, Whole-brain radiotherapy, Radiotherapy boost, Neurological symptoms

## Abstract

**Purpose:**

Stereotactic radiosurgery (SRS) has become a standard approach for the treatment of patients with few metastatic brain lesions. However, the optimal treatment approach for the use radiotherapy in the treatment of non-small cell lung cancer (NSCLC) patients with brain metastases (BMs) remain unclear. This study aimed to compare the survival outcomes and intracranial local control in NSCLC patients with 1–4 BMs who are treated with SRS using linear accelerators (LINAC-SRS), whole-brain radiotherapy (WBRT), or WBRT plus radiotherapy boost (WBRT + RTB).

**Materials and methods:**

We retrospectively analyzed 156 NSCLC patients with 1–4 BMs who received LINAC-SRS, WBRT, and WBRT + RTB. The median overall survival (OS), intracranial progression-free survival (iPFS), and distant brain failure-free survival (DBF-FS) and related prognostic factors were analyzed.

**Results:**

The median follow-up period was 31.6 months. The median OS times in the LINAC-SRS, WBRT, and WBRT + RTB groups were not reached, 33.3 months and 27.9 months, respectively. The difference in survival rate was non-significant (*P* = 0.909). The 2-year iPFS and DBF-FS rates in the LINAC-SRS, WBRT and WBRT + RTB groups were 51.6% and 37.5%; 42.0% and 50.4%; and 51.1% and 56.1%, respectively. There was no significant difference in 2-year iPFS or DBF-FS among the three groups (*P* = 0.572 for iPFS*, P* = 0.628 for DBF-FS). Multivariate analysis showed that the independent adverse prognostic factors for OS, iPFS, and DBF-FS were neurological symptoms, recursive partitioning analysis (RPA) class, and targeted therapy.

**Conclusion:**

LINAC-SRS did not result in significantly superior survival times or intracranial local control compared to WBRT or WBRT + RTB in the treatment of NSCLC patients with 1–4 BMs.

**Supplementary Information:**

The online version contains supplementary material available at 10.1186/s12885-022-10083-8.

## Introduction

Lung cancer remains the leading cause of cancer-related death, and an estimated 1.8 million deaths were recorded in 2020 [1]. Non-small cell lung cancer (NSCLC) accounts for approximately 85% of lung cancer cases. Approximately 25% of patients with advanced NSCLC have brain metastases (BMs) at diagnosis. A total of 40–50% of NSCLC patients develop BMs during the course of the disease [2]. BM is one of the key causes of death in NSCLC patients [3]. With the increased incidence and earlier diagnosis of BMs, there is a growing need to improve the prognosis of these patients through the use of individual treatment solutions.

Radiotherapy (RT) is key part for BM management, either alone or combined with surgery and systemic therapies. Intracranial radiotherapy (iRT), includes whole-brain radiation therapy (WBRT), stereotactic radiosurgery (SRS, which refers to both single-fraction and multi-fraction stereotactic radiation treatments), and WBRT with integrated boost. The type of intracranial RT that is used depends on the tumor characteristics, patient general status and medical condition. Historically, WBRT has long been a mainstay of brain metastasis treatment, and it provides symptom relief and prolongs survival. More recently, SRS has become a standard approach for patients with oligometastatic BMs because, when it is compared with WBRT or SRS plus WBRT, it causes less damage to neurocognitive function and achieves similar intracranial control [4]. However, because of the high heterogeneity of NSCLC, and the substantial differences in medical conditions and economic levels in different regions, WBRT with or without simultaneous integrated boost still plays an important role in the radiotherapy of brain metastasis. Moreover, in the era of targeted therapy and immune therapy, it is more difficult to choose individualized treatment approaches.

The aim of this real-world retrospective study was to reassess differences in the survival and intracranial control of NSCLC patients with 1–4 BM lesions who were treated with LINAC-SRS, WBRT, or WBRT + RTB in order to aid in the clinical considerations involved in selecting an optimal RT strategy.

## Materials and methods

### Study design and patients

We retrospectively reviewed the medical records of NSCLC patients with 1–4 BMs at the Third Affiliated Hospital of Kunming Medical University (Yunnan Cancer Hospital, Kunming, China) between May 2010 and August 2019.

The eligibility criteria were as follows:

1. patients aged ≥ 18 years;
2. patients with cytologically or histologically proven NSCLC (squamous cell carcinoma or adenocarcinoma);
3. patients with BMs that were confirmed by gadolinium-enhanced magnetic resonance imaging (MRI) or contrast-enhanced CT that showed single or multiple lesions (1–4 BMs) in the brain;
4. patients treated with brain radiotherapy;
5. patients with sufficient available information.

Patients were excluded if they had interrupted treatment for more than 1 week during intracranial RT, had a previous history of craniocerebral radiotherapy, or had incomplete information regarding the radiation dose and imaging results. The protocol was reviewed and approved by the Ethics Committee of the Third Affiliated Hospital of Kunming Medical University. Informed consent was waived by the committee because of the retrospective nature of this study. This trial was conducted in accordance with the Declaration of Helsinki. In total, 156 patients were eligible for this study (Seen in additional file 1.Trial profile.).

Clinical and treatment data, including sex, age, Karnofsky Performance Scale (KPS) score, smoking history, histology, BM number, maximum brain lesion diameter, primary lesion status, targeted therapy after BMs detection, extracranial metastasis (EM) status at the BMs were confirmation, neurological symptoms, initial treatment of BMs at lung cancer diagnosis, chemotherapy, radiotherapy information, recursive partitioning analysis (RPA) data, and graded prognostic assessment (GPA) were recorded.

### Radiation treatment planning and delivery

A total of 156 patients were eligible for this study, and 79 patients underwent WBRT with a median dose of 40 Gy/20F (range, 16–54 Gy/8-25F, 5 F/week). Among the patients, 22 (27.8%) received 30 Gy/10F, 29 (36.7%) received 40 Gy/20F, 9 (11.4%) received 46 Gy/23F, 6 (7.6%) received 36 Gy/18F, and 13 (16.5%) received a median dose of 43.2 Gy/23F. Among the 79 patients, based on the judgment of radiation oncologists and according to the tumor volume, tumor location, and neurological symptoms, 35 patients (32%) underwent concurrent or sequential RT of local lesions (WBRT plus focal radiation boost, RTB) with a median dose of 11 Gy (range, 6–21.6 Gy). The PTV boost was defined as a 3 mm margin to the GTV boost. Of these 35 patients, 10 (28.6%) received concurrent RTB, and 25 (71.4%) received sequential RTB.

A total of 77 NSCLC patients with BMs were treated with LINAC-SRS under tomotherapy or volumetric modulated arc therapy (VMAT) technology. All the patients underwent brain gadolinium-enhanced MRI scanning and head-neck-shoulder modeling, in addition to contrast-enhanced localization CT scanning while the head model and body frame were fixed, before RT. Image fusion for the enhanced MRI and enhanced localization CT images was completed on a Pinnacle planning system. The gross tumor volume (GTV) was delineated on the fused localization CT, and the planning target volume (PTV) consisted of the GTV of all metastases with a margin of 3 mm surrounding each metastasis. The prescribed dose ensured at least 95% PTV and 100% GTV target coverage.

### Follow-ups and endpoints

For patients who underwent regular admission, evaluation of whether the patients with BMs relapsed, developed new disease or died was performed with the case-patient imaging retrieval system, and this evaluation included a review of the results of contrast-enhanced MRI or contrast-enhanced CT of the head 1 month after the end of RT and every 3 months thereafter. For patients who could not be followed up through the case and outpatient system, information about whether the BMs had recurred or were newly diagnosed and whether the patient had died was obtained by telephone follow-up. The intracranial response and systemic response were assessed by Response Assessment in Neuro-Oncology Brain Metastases (RANO-BM) criteria and Response Evaluation Criteria In Solid Tumours (RECISTv1.1), and these responses were classified as complete response (CR), partial response (PR), stable disease (SD), and progressive disease (PD) [5, 6]. Overall survival (OS) was calculated from the day of BM diagnosis to death or the last day of follow-up. Intracranial progression-free survival (iPFS) was defined as the time from the end of brain RT to the progression of previously treated brain lesions or the last day of follow-up. Distant brain failure-free survival (DBF-FS) time was defined as the time from the completion of intracranial RT to the day development of one or more new brain metastases outside of the previous target volume or the last day of follow-up. Adverse events (AEs) were evaluated and graded based on the National Cancer Institute Common Terminology Criteria for Adverse Events (CTCAE) version 4.0. Patients were tested for memory and cognitive decline using the Simple Mini-Mental State Examination (MMSE). At the follow-up date of August 31, 2020, the endpoint event was death, whereas those who were lost to follow-up or who were alive at this date were censored.

### Statistical analyses

To compare the baseline characteristics among the three groups of patients, the chi-square test (x^2^ test) or Fisher's exact test was used for categorical variables. Survival curves were generated using the Kaplan–Meier method and compared using the log-rank test. The log-rank test was also used for univariate analyses of prognostic factors. Variables with *P* values < 0.1 in the univariate analyses were further analyzed in multivariate analyses using the Cox proportional hazards regression model to assess prognostic factors for OS, iPFS, and DBF-FS. The tests were two-sided, and *P* < 0.05 was considered statistically significant. The statistical analyses were conducted with SPSS software (version 25.0; IBM Corp, Armonk, NY, USA).

## Results

### Baseline characteristics of the patients

A total of 156 NSCLC patients with metastases were divided into three groups according to the craniospinal radiotherapy method used: the LINAC-SRS group, the WBRT alone (WBRT) group, and the WBRT + RTB group. The patient characteristics are described in Table 1. For the entire cohort, the median age was 55 years (range, 22–78 years). The majority of patients (85.9%) had a pathological type of adenocarcinoma. The KPS score was centered at 70–90, and 14 patients (9.0%) had a KPS score ≤ 70. Sixty-six patients (42.3%) had BMs that were detected at the time of initial NSCLC diagnosis, 69 patients (44.2%) had definite neurological symptoms such as dizziness, headache, nausea, and vomiting, 91 patients (58.3%) had only one cranial metastasis, and 130 patients (83.3%) had metastases with a maximum diameter (Dmax) ≤ 3 cm. Thoracic surgery was performed in 68 patients (43.6%). More than half of the patients (80.1%) had primary lesions in the lungs that were stable. Concurrent chemotherapy with a platinum-based regimen consisting of cranial RT was administered to 42 (26.9%) patients, and 47 (30.1%) patients received targeted therapy (including EGFR-TKIs, lapatinib, bevacizumab, and anlotinib) after BM diagnosis. There were 2 (1.28%) patients received immuotherapy (Camrelizumab, Sintilimab).

**Table 1:** Patients characteristics according to the treatment group

Characteristics	All(%)	LINAC-SR(%)	WBRT(%)	WBRT + RTB (%)	*P*
Number of patients	156(100)	77(49.4)	44(28.2)	35(22.4)	
Sex					0.350
Female	58(37.2)	33(42.9)	14(31.8)	11(31.4)	
Male	98(62.8)	44(57.1)	30(68.2)	24(68.6)	
Age, years					0.745
≤ 50	57(36.5)	30(39.0)	16(36.4)	11(31.4)	
≥ 51	99(63.5)	47(61.0)	28(63.6)	24(68.6)	
Smoking history					0.140
No	76(48.7)	34(44.2)	27(61.4)	15(42.9)	
Yes	80(51.3)	43(55.8)	17(38.6)	20(51.7)	
KPS scores					0.213
≥ 90	96(61.5)	46(59.7)	28(63.6)	22(62.9)	
≤ 80	60(38.5)	31(40.3)	16(36.4)	13(37.1)	
Tumor histology					0.056
Squamous cell carcinoma	22(14.1)	16(20.8)	4(9.1)	2(5.7)	
Adenocarcinoma	134(85.9)	61(79.2)	40(90.9)	33(94.3)	
Thoracic operation					0.784
Yes	68(43.6)	32(41.6)	19(43.2)	17(48.6)	
No	88(56.4)	45(58.4)	25(56.8)	18(51.4)	
Initial treatment of BMs ^a^					0.628
Yes	66(42.3)	30(39.0)	19(43.2)	17(48.6)	
No	90(57.7)	47(61.0)	25(56.8)	18(51.4)	
Neurologic symptoms					0.613
Yes	69(44.2)	32(41.6)	19(43.2)	18(51.4)	
No	87(55.8)	45(58.4)	25(56.8)	17(48.6)	
Number of BMs					0.758
1	91(58.3)	45(58.4)	24(54.5)	22(62.9)	
2–4	65(41.7)	32(41.6)	20(45.5)	13(37.1)	
BM size, Dmax (cm)					0.167
≤ 3	130(83.3)	68(88.3)	33(75.0)	29(82.9)	
> 3	26(16.7)	9(11.7)	11(25.0)	6(17.1)	
Primary disease control					0.003
Yes	125(80.1)	70(90.9)	29(65.9)	26(74.3)	
No	31(19.9)	7(9.1)	15(34.1)	9(25.7)	
EMs					0.985
Yes	76(48.7)	38(49.4)	21(47.7)	17(48.6)	
No	80(51.3)	39(50.6)	23(52.3)	18(51.4)	
RPA class					0.902
1	52(33.3)	27(35.1)	14(31.8)	11(31.4)	
2	104(66.7)	50(64.9)	30(68.2)	24(68.6)	
GPA scores					0.411
0.5–1.5	22(14.1)	8(10.4)	8(18.2)	6(17.1)	
2–2.5	62(39.7)	33(42.9)	19(43.2)	10(28.6)	
≥ 3	72(46.2)	36(46.8)	17(38.6)	19(54.3)	
Concurrent chemotherapy					0.294
Yes	42(26.9)	25(32.5)	10(22.7)	7(20.0)	
No	114(73.1)	52(67.5)	34(77.3)	28(80.0)	
TT after BMs					0.091
Yes	47(30.1)	17(22.1)	16(36.4)	14(40.0)	
No	109(69.9)	60(77.9)	28(63.6)	21(60.0)	

*BMs* brain metastases, *EMs* extracranial metastases, *GPA* graded prognostic assessment, *HR* hazard ratio, *LINAC-SRS* stereotactic radiotherapy using linear accelerators, *KPS* Kanofsky Performance Scale, *RPA* recursive partitioning analysis, *TT* targeted therapy, *WBRT* whole-brain radiotherapy, *WBRT* + *RTB* whole-brain radiotherapy plus radiotherapy boost

Among the three groups, there were more patients with stable primary disease in the lung were observed in the LINAC-SRS group than in the WBRT and WBRT + RTB groups (56.0% vs. 23.2% vs. 20.8%, *P* = 0.003). Other clinical features, including sex, age, smoking status, KPS score, pathological type, EMs, thoracic operation, neurological symptoms, GPA scores, RPA class, maximum brain lesion diameter, concurrent chemotherapy and targeted therapy after BM diagnosis, were well-matched among the three treatment groups (all *P* > 0.05, Table 1).

### Overall survival

The median follow-up time was 31.6 months (range, 0.5–98.4 months). By the last follow-up visit, 60 patients (38.4%) had died, including 26 patients in the LINAC-SRS group, 22 patients in the WBRT group, and 12 patients in the WBRT + RTB group.

**Fig. 1:**
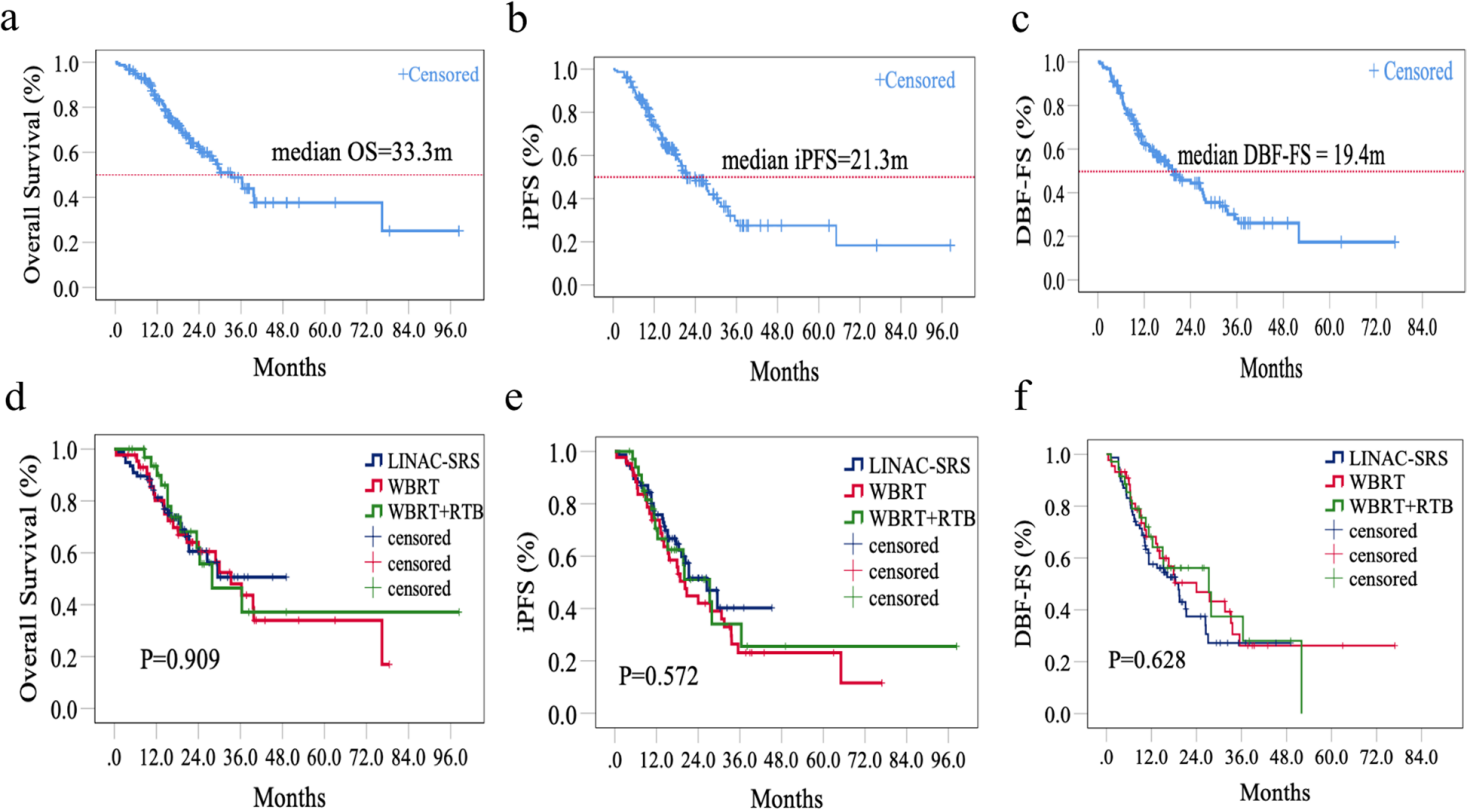
Cumulative incidence of OS, iPFS, and DBF-FS for all patients (**a**, **b**, **c**) and comparison of cumulative incidence of OS, iPFS, and DBF-FS in three groups (**d**, **e**, **f**). Abbreviations: LINAC-SRS, stereotactic radiotherapy based on linear accelerators; OS, overall survival; iPFS, intracranial progression-free survival; DBF-FS, distant brain failure-free survival; m, months; RTB, radiotherapy boost; WBRT, whole-brain radiotherapy

For the entire cohort, the median OS was 33.3 months, and the 6-, 12-, and 24-month OS rates were 94.8%, 83.7%, and 59.9%, respectively (Fig. 1a). The median OS was not reached in the LINAC-SRS group, the mOS in the WBRT group was 33.3 months, and that in the WBRT + RTB group was 27.9 months. The 6-, 12-, and 24-month OS rates in the LINAC-SRS group were 90.9%, 81.3%, and 60.6%, those in the WBRT alone group were 97.7%, 80.2%, 60.5%, and those in the WBRT + RTB group were 100%, 93.4%, 55.7%, respectively (*P* = 0.909; Fig. 1d).

Univariate and multivariate analyses were performed to determine the prognostic indicators for OS (Tables 2 and 3). The univariate analysis revealed that female sex (*P* = 0.018), no smoking history (*P* = 0.033), adenocarcinoma (*P* = 0.023), absence of neurological symptoms (*P* < 0.000), RPA class 1 (*P* < 0.000), and BMs with a Dmax ≤ 3 cm (*P* = 0.012) were significantly associated with better OS.

**Table 2:** Univariate analysis of overall survival

Parameter	OS (months)	P	Parameter	OS (months)	P
Sex		0.018	Primary disease control		0.277
Female	-		Yes	36.3	
Male	33.3		No	24.0	
Age, years		0.159	Extracranial metastases		0.094
≤ 50	76.4		No	36.4	
≥ 51	29.5		Yes	28.9	
RPA class		< 0.000	Number of BMs		0.612
1	76.4		1	39.6	
2	24.0		2–4	29.5	
GPA scores		0.216	BMsize, Dmax(cm)		0.012
0.5–1.5	28.9		≤ 3	36.4	
2–2.5	29.5		> 3	18.4	
≥ 3	76.4		Neurologic symptoms		< 0.000
Smoking history		0.033	No	76.4	
N0	39.8		Yes	21.3	
KPS scores		0.166			
≥ 90	39.8				
≤ 80	28.9		TT after BMs		0.101
Tumor histology		0.023	Yes	39.6	
SCC	21.3		No	30.0	
Adenocarcinoma	36.3		Initial treatment of BMs^a^		0.666
Thoracic operation		0.084	Yes	27.9	
Yes	39.8		No	36.4	
No	28.9

**Table 3:** Multivariate analysis of OS

Parameter	median OS (m)	HR	95%CI	*P*
Sex				0.111
Female	-	1.000		
Male	33.3	1.976	0.856–4.558	
Smoking history				0.696
N0	39.8	1.000		
Yes	27.9	1.154	0.562–2.369	
Tumor histology				0.175
SCC	21.3	1.000		
Adenocarcinoma	36.3	0.612	0.301–1.244	
Thoracic operation				0.966
Yes	39.8	1.000		
No	28.9	1.013	0.562–1.825	
Neurologic symptoms				0.002
No	76.4	1.000		
Yes	21.3	2.462	1.401–4.326	
BMsize, Dmax(cm)				0.189
≤ 3	36.4	1.000		
> 3	18.4	1.555	0.805–3.004	
EMs				0.581
No	36.4	1.000		
Yes	28.9	1.190	0.642–2.207	
RPA class				0.010
1	76.4	1.000		
2	24.0	2.918	1.294–6.579	

Abbreviations: *BMs* brain metastases, *EMs* extracranial metastases, *GPA* graded prognostic assessment, *HR* hazard ratio, *LINAC-SRS* stereotactic radiotherapy using linear accelerators, *KPS* Kanofsky Performance Scale, *RPA* recursive partitioning analysis, *SCC* squamous cell carcinoma, *TT* targeted therapy, *WBRT* whole-brain radiotherapy, *WBRT* + *RTB* whole-brain radiotherapy plus radiotherapy boost

The multivariate analysis found that RPA class 2 [hazard ratio (HR): 2.918, 95% confidence interval (CI): 1.294–6.579, *P* = 0.010] and neurological symptoms (HR: 2.462, 95% CI: 1.401–4.326, *P* = 0.002) were independent factors associated with worse OS. The mOS of patients without neurological symptoms was significantly longer than that of patients with neurological symptoms, and patients without neurological symptoms had a surprising median OS of 76.4 months.

### Intracranial progression-free survival

In all patients, the median iPFS was 21.3 months, and the 1- and 2-year iPFS rates were 74.2% and 48.3%, respectively (Fig. 1b). The median iPFS was 26.5 months in the LINAC-SRS group, 20.2 months in the WBRT group and 27.3 months in the WBRT + RTB group. The 1- and 2-year iPFS rates were 75.8% and 51.6% in the LINAC-SRS group, 73.8% and 42.0% in the WBRT group and 70.6% and 51.1% in the WBRT + RTB group, respectively. There was no significant difference among the three groups (*P* = 0.572; Fig. 1e).

The univariate analysis revealed that thoracic surgery (*P* = 0.020), absence of neurological symptoms (*P* < 0.000), intracranial metastases with Dmax ≤ 3 cm (*P* = 0.008), and RPA class 1 (*P* = 0.009) were associated with a significantly better iPFS. Multivariate analysis indicated that the independent adverse prognostic factors for iPFS were the absence of neurological symptoms (HR: 2.025, 95% CI: 1.238–3.313, *P* = 0.005) and the absence of BMs without having undergone targeted therapy ( HR: 2.073, 95% CI: 1.221–3.520, *P* = 0.007, Tables 4 and 5).

**Table 4:** Univariate analysis of iPFS

Parameter	iPFS(m)	*P*	Parameter	iPFS(m)	*P*
Sex		0.538	Number of BMs		0.490
Female	21.3		1	27.5	
Male	24.0		2–4	20.3	
Age, years		0.129	BM size, Dmax(cm)		0.008
≤ 50	31.5		≤ 3	27.9	
≥ 51	20.3		> 3	18.2	
Smoking history		0.501	Primary disease control		0.880
No	20.7		Yes	21.3	
Yes	21.3		No	24.0	
KPS scores		0.998	EMs		0.226
≥ 90	21.5		No	27.9	
≤ 80	21.3		Yes	20.0	
Tumor histology		0.168	Concurrent chemotherapy		0.407
SCC	22.2		Yes	21.3	
Adenocarcinoma	20.7		No	21.3	
Thoracic operation		0.020	TT after BMs		0.065
Yes	30.7		Yes	26.5	
No	18.8		No	20.3	
Initial treatment of BMs		0.234	GPA scores		0.108
Yes	27.9		0.5–1.5	18.8	
No	36.4		2–2.5	19.7	
Neurologic symptoms		< 0.00	≥ 3	31.5	
No	30.7				
Yes	17.6				
RPA class		0.009			
1	33.5				
2	19.7

**Table 5:** Multivariate analysis of iPFS

Parameter	iPFS (months)	HR	95%CI	*P*
Neurologic symptoms				0.002
No	30.7	1.000		
Yes	17.6	2.025	1.238-3.313	
BM size, Dmax(cm)				0.213
≤ 3	27.9	1.000		
> 3	18.2	1.458	0.806–2.639	
RPA class				0.055
1	33.5	1.000		
2	19.7	1.710	0.989–2.957	
TT after BMs				0.007
Yes	26.5	1.000		
No	20.3	2.073	1.221–3.520	

*BMs* brain metastases, *EMs* extracranial metastases, *GPA* graded prognostic assessment, *HR* hazard ratio, *iPFS* intracranial progression-free survival, *LINAC-SRS* stereotactic radiotherapy using linear accelerators, *KPS* Kanofsky Performance Scale, *RPA* recursive partitioning analysis, *SCC* squamous cell carcinoma, *TT* targeted therapy, *WBRT* whole-brain radiotherapy, *WBRT* + *RTB* whole-brain radiotherapy plus radiotherapy boost

### Distant brain failure-free survival

The median DBF-FS among all the patients was 19.4 months, and the 1- and 2-year DBF-FS rates were 62.7% and 44.4%, respectively (Fig. 1c). The median DBF-FS was 19.2 months in the LINAC-SRS group, 24.0 months in the WBRT group and 27.3 months in the WBRT + RTB group. The 1- and 2-year DBF-FS rates were 57.6% and 37.5% in the LINAC-SRS group, 68.2% and 50.4% in the WBRT group and 68.2% and 56.1% in the WBRT + RTB group, respectively. Although the 2-year DBF-FS rate was not significantly different among the groups, the risk of the development of new BMs was higher in the LINAC-SRS group (*P* = 0.628, Fig. 1f).

In the univariate analysis, the absence of neurological symptoms (*P* = 0.002), RPA class 1 (*P* = 0.009), and BMs with targeted therapy (*P* = 0.046) were predictors of significantly better DBF-FS (Additional file 2.1). Multivariate analysis indicated that the absence of neurological symptoms (HR: 1.874, 95% CI: 1.189–2.954, *P* = 0.007), RPA class 2 (HR: 1.812, 95% CI: 1.094–3.000, *P* = 0.021), and no history of targeted therapy (HR: 1.932, 95% CI: 1.164–3.206, *P* = 0.011) were independent adverse prognostic factors for DBF-FS (Additional file 2.2).

### Subgroup analysis of patients with and without neurological symptoms

**Fig. 2:**
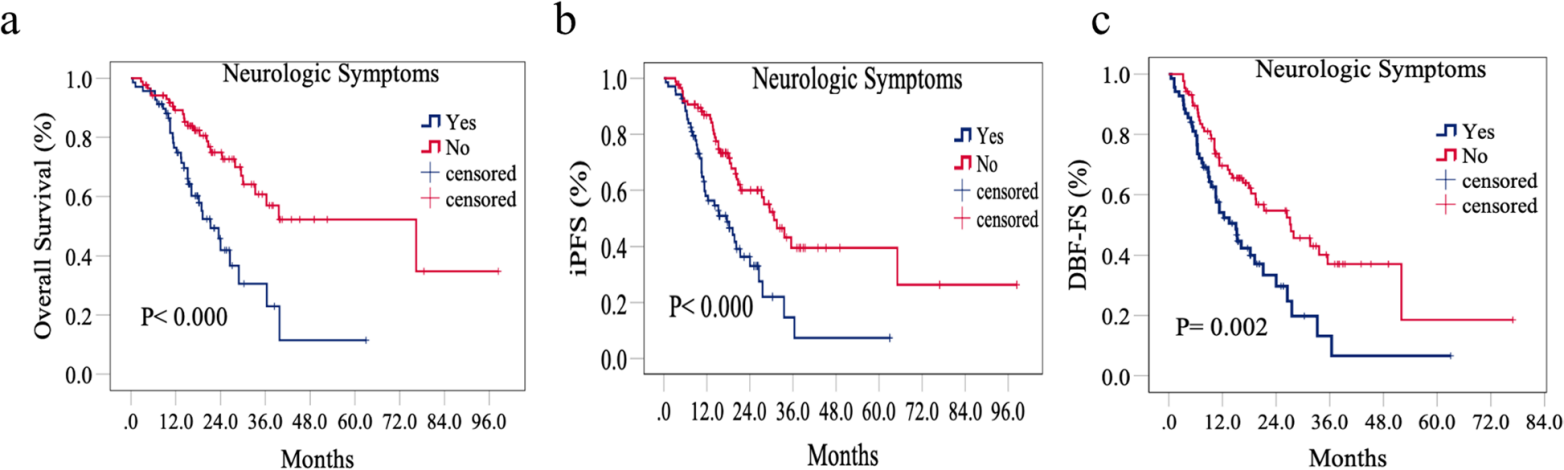
Comparison of cumulative incidence of OS (**a**), iPFS (**b**), DBF-FS (**c**) of patients with or without neurological symptoms

In this study, 87 patients had neurological symptoms, and 69 patients lacked neurological symptoms. The median OS of patients without neurological symptoms was up to 76.4 months, and the median iPFS and DBF-FS were 30.7 months and 27.3 months, respectively, which were significantly longer than those in patients with neurological symptoms (21.3 months, 19.2 months, and 15.0 months, respectively, Fig. 2).

Based on the results of the univariate and multivariate analyses, we concluded that the absence of neurological symptoms was an independent prognostic factor associated with OS, iPFS, and DBF-FS. Therefore, we performed an additional subgroup analysis of patients with and without neurological symptoms.

### Subgroup analysis of patients with brain metastases and neurological symptoms

Among all the patients, 69 patients had neurological symptoms, of whom 32 patients (46.4%) were treated with LINAC-SRS, 19 patients (27.5%) were treated with WBRT alone, and 18 patients (26.1%) were treated with WBRT + RTB. The baseline characteristics were well balanced, except for the size of the brain lesions, among the LINAC-SRS, WBRT, and WBRT + RTB groups. There were more patients with Dmax ≤ 3 cm intracranial metastases in the LINAC-SRS group than in the WBRT and WBRT + RTB groups (54.2% vs. 18.8% vs. 27.1%, *P* = 0.038, Additional file 3).

**Fig. 3:**
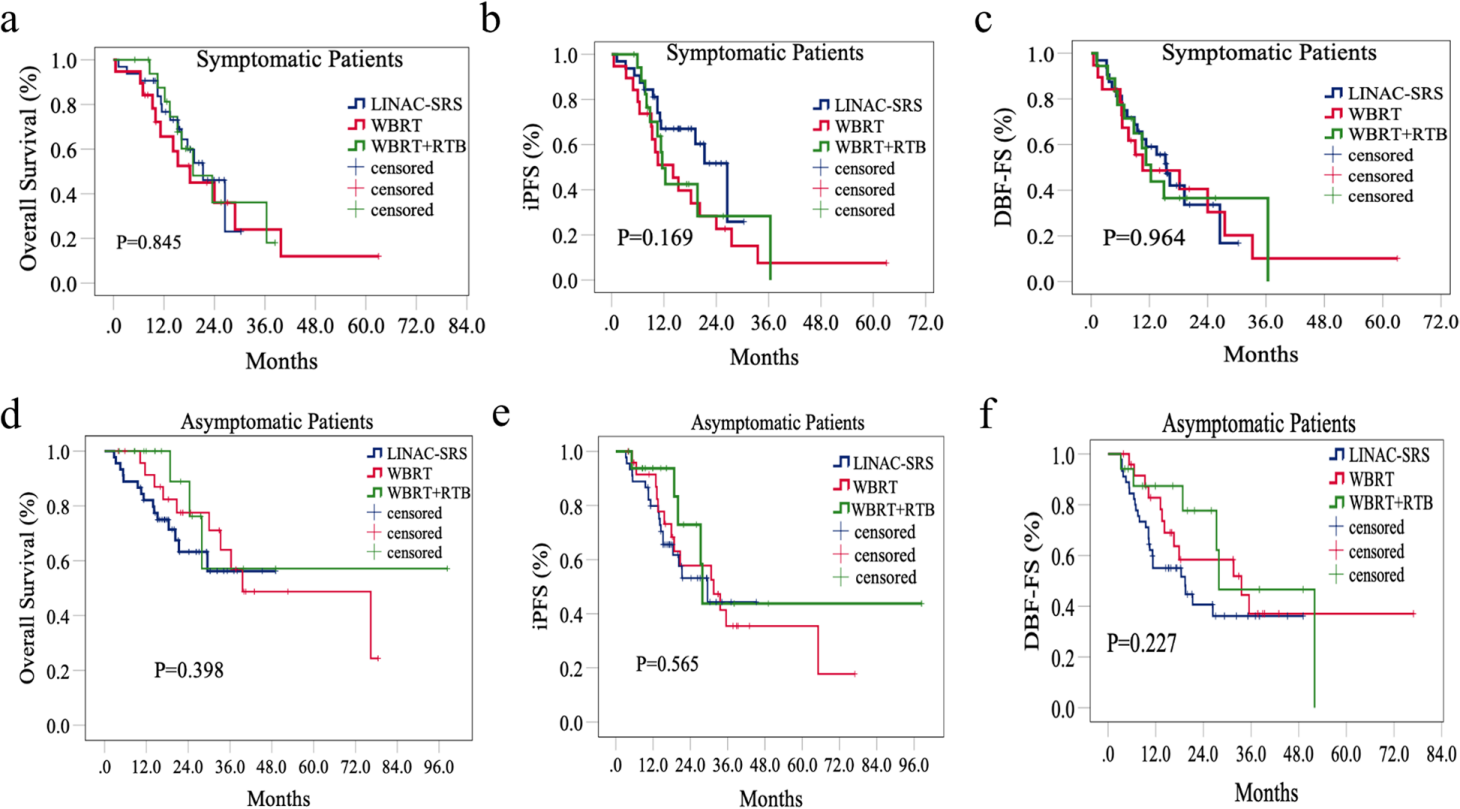
Comparison of cumulative incidence of OS, iPFS, DBF-FS for patients with neurological symptoms (**a**, **b**, **c**) and for asymptomatic patients (**d**, **e**, **f**) among LINAC-SRS, WBRT group and WBRT + RTB groups. Abbreviations: LINAC-SRS, stereotactic radiotherapy based on linear accelerators; OS, overall survival; iPFS, intracranial progression-free survival; DBF-FS, distant brain failure-free survival; m, months; RTB, radiotherapy boost; WBRT, whole-brain radiotherapy

The median OS was 21.6 months in the LINAC-SRS group, 18.2 months in the WBRT group, and 18.9 months in the WBRT + RTB group at the last follow-up. There was no difference in median OS among the three treatment groups (*P* = 0.845, Fig. 3a). The median iPFS was 26.5 months in the LINAC-SRS group, 14.1 months in the WBRT group, and 11.6 months in the WBRT + RTB group (*P* = 0.169, Fig. 3b). No significant difference in median DBF-FS was observed among the three groups (15.5 months in the LINAC-SRS group, 10.6 months in the WBRT group, and 12.3 months in the WBRT + RTB group, *P* = 0.964, Fig. 3c).

### Subgroup analysis of patients with brain metastases without neurological symptoms

We also performed a subgroup analysis of patients who did not have neurological symptoms. Of the eighty-seven patients without neurological symptoms, 45 patients (51.7%) were treated with LINAC-SRS, 25 patients (28.8%) were treated with WBRT alone, and 17 patients (19.5%) were treated with WBRT + RTB. Patients with stable primary lesions were more likely to receive LINAC-SRS than WBRT or WBRT + RTB (93.3% vs. 56.0% vs. 82.4%, *P* = 0.001). There were more patients treated with concurrent chemotherapy in the LINAC-SRS group than in the WBRT and WBRT + RTB groups (33.3% vs. 12.0% vs. 5.9%, *P* = 0.023). Most (93.1%) of the asymptomatic patients started iRT within 5 months after diagnosis of BM, and all patients received iRT within 10 months after BM detection (Additional file 4).

Among the 87 patients without neurological symptoms, the median OS, iPFS, and DBF-FS were 76.4 months, 30.7 months, and 27.3 months, respectively. There was no difference in median OS among the three treatment groups (mOS in the LINAC-SRS and WBRT + RTB groups had not yet been reached, 39.6 months in the WBRT group, *P* = 0.398, Fig. 3d). The median iPFS was 29.5 months in the LINAC-SRS group, 31.5 months in the WBRT group and 27.9 months in the WBRT + RTB group (*P* = 0.565, Fig. 3e). Compared with WBRT and WBRT + RTB, LINAC-SRS was also not beneficial in terms of median DBF-FS, and a significant difference in median DBF-FS was not observed among the three groups (19.4 months in the LINAC-SRS group, 33.6 months in the WBRT group, and 27.9 months in the WBRT + RTB group, *P* = 0.227, Fig. 3f).

### Comparison of the adverse reactions of different intracranial radiotherapies

The poor status of the nervous system in the patients of the 3 groups mainly manifested as ncephaledema (nausea, vomitus, dizziness and so on), memory decline, and cognitive impairment. No grade 3 to 5 toxicities were reported.

There was no significant difference among the 3 groups in terms of encephaledema, memory or cognitive decline after iRT. The incidence rate of memory and cognitive decline in group LINAC-SRS, group WBRT, and group WBRT + RTB was respectively 20.5%, 20.77%, and 18.18% (*P* = 0.108). There was one case of grade 2 toxicity (motor neuropathy, depressed level of consciousness) attributed to radiation treatment in WBRT and WBRT + RTB. No significant difference was found in nervous system toxicity degree ( *P* = 0.702). The incidence rate of cutaneous radiation reaction in LINAC-SRS, WBRT and WBRT + RTB group was 36.36%, 13.64%, and 40.0%, respectively (*P* = 0.156). The most common systemic toxicity were digestive system side-effect and myelosuppression (3.9%, 4.55%,  and 2.86% in LINAC-SRS, WBRT and WBRT + RTB group, respectively, *P* = 0.738, Table 6).

**Table 6:** The main toxicity attributed to intracranial radiotherapy

Toxicity	Total (*N* = 156)	LINAC-SRS (*N* = 77)	WBRT (*N* = 44)	WBRT + RTB (*N* = 35)	*P*
Nervous system (encephaledema, memory and cognitive decline N (%)	32 (20.5)	16 (20.77)	8 (18.18)^a^	8 (22.86)^a^	0.108
Cutaneous radiation reaction N (%)	48 (30.8)	28 (36.36)	6 (13.64)	14 (40.00)	0.156
Systemic toxicity^b^ N (%)	6 (3.85)	3 (3.90)	2 (4.55)	1 (2.86)	0.738

^a^There was one case of grade 2 toxicity

^b^The most common systemic toxicities were digestive system side-effect and myelosuppression. *LINAC-SRS* stereotactic radiotherapy using linear accelerators, *WBRT* whole-brain radiotherapy, *WBRT* + *RTB* whole-brain radiotherapy plus radiotherapy boost

## Discussion

BMs are a common, severe manifestation that occur in 20–50% of patients with NSCLC. BMs negatively impact the patient’s quality of life and result in a shorter lifespan. The aim of treatments in these patients is to control local progression, prolong OS and relieve neurological symptoms. Currently, therapies for NSCLC patients with 1–4 BMs include RT, targeted therapy, surgery, chemotherapy, and immunotherapy. These treatments often used sequentially or in combination, contribute to longer survival of patients with BMs. However, the best treatment strategy for BMs is controversial.

RT is emerging as one of the most promising treatments for patients with BMs. SRS has gradually become the main RT approach in patients with 1–4 BM sites, especially in developed countries and institutions. Hypofractionated irradiation (> 2 Gy per fraction) has been reported to exert antitumor immune effects. However, not all patients with 1–4 intracranial sites are suitable for SRS due to large tumor size, dangerous tumor location, unstable primary lesion, and so on. Traditional WBRT or WBRT plus BOOST is still used due to the compliance of actual BM patients [7] Therefore, we analyzed differences in the survival and intracranial control of NSCLC patients with 1–4 BM lesions who treated with LINAC-SRS, WBRT and WBRT + RTB in our center in the past 10 years. We also searched for prognostic markers that could aid in clinical decision-making.

In this study, we retrospectively analyzed the clinical data of 156 NSCLC patients with 1–4 site BMs who were treated with brain RT at our institution. The median OS for all patients was 33.3 months, and the median iPFS was 21.3 months, which was better than that in multiple previous reports [7, 4]. The median OS and iPFS associated with WBRT + SIB (simultaneous integrated boost) were 24.3 months and 9.1 months, respectively, in a report by another Chinese institution [8]. The improved survival outcome and local control may be attributed to the high percentage (85.9%) of pathological type (adenocarcinoma), more than half of patients having only one BM lesion, nearly all LINAC-SRS doses being ≥ 30 Gy in daily fractions, and the use of multidisciplinary comprehensive treatments (targeted therapy and chemotherapy). Adenocarcinoma, confirmed by histology, was associated with better survival outcomes than squamous cell carcinomas [9]. Myrehaug, S et al. [10] reported that better local control was achieved with hypofractionated SRT at a dose ≥ 30 Gy than at a dose < 30 Gy. A prospective phase 3 trial (NCCTG N107C [Alliance]/CEC.3) also found that for patients with BMs where WBRT is recommended, shorter course hypofractionated regimens (30 Gy/10F) remain the current standard of care [11].

The most important finding in this study was that no superior median OS, iPFS, or DBF-FS was observed in the LINAC-SRS group compared to the WBRT and WBRT + RTB groups. These results partly challenged the current guidelines for BM management in patients with NSCLC. The National Comprehensive Cancer Network (NCCN) guidelines version 7·2021 recommend SRS alone or surgery followed by WBRT or SRS for patients with few BMs. Our results are consis with data from many studies, including randomized clinical trials (RCTs) [11, 12] and matched-pair studies [13], with similar OS observed in patients with 1–3 BMs who were treated with WBRT and SRT. However, another multi-institutional, retrospective study (Miller,Kotecha et al. 2016) concluded that patients with ≤ 4 BMs and tumor size ≤ 4 cm who were treated with SRS had better survival than those treated with WBRT.

Our previous study [7] found that WBRT + RTB significantly improved iPFS compared with WBRT alone, in driver gene-negative patients. However, half of the patients (53.9%) had ≥ 3 BMs. A population-based study of 2140 NSCLC patients showed that OS after RT for BMs improved during 2006–2018 only in patients treated with SRT, not WBRT, suggested that SRS alone was the preferred strategy in patients with few BMs due to its similar OS and less cognitive impairment than WBRT + SRS [14]. However, there may be bias because only the fittest patients are referred for SRT, which is common in many studies. Notably, although the 2-year DBF-FS rate was not significantly different among the groups, the risk of new BMs was higher in the LINAC-SRS group. Meng Ni et al. [15] reported that patients who were treated with WBRT + boost achieved significantly longer OS than those treated with SRS (WBRT + boost vs. SRS, 22.2 vs. 17.3 months, *P* = 0.011). A multidisciplinary approach is strongly recommended to personalize the treatment of each patient and improve the therapeutic ratio.

We also found that receiving targeted therapy for BMs was a significant predictor of better DBF-FS. BM management is controversial in NSCLC patients who are EGFR- or ALK-positive. Patients with EGFR mutations tend to have a particularly high incidence of BMs (approximately 70%) [16]. However, data on the OS benefits of WBRT/SRS compared to target agents are conflicting, and this approach is associated with significant loss of neurocognitive function. Newer target agents with improved CNS efficacy have challenged the use of early radiotherapy in NSCLC patients with oncogenic driver mutations. Another retrospective real-world study showed that patients with EGFR-mutated NSCLC who underwent intracranial local therapy achieved better survival [17]. Interestingly, in advanced lung adenocarcinoma patients with EGFR mutations and asymptomatic BM, brain radiotherapy in combination with tyrosine kinase inhibitors (TKIs) could result in better iPFS and OS(iPFS, 21.5 vs. 14.8 months, *P* = 0.026; mOS, 36 vs. 23 months, *P *= 0.041) than TKI alone [18].However, another multi-institutional retrospective analysis found no significant differences between treatment with CNS-penetrant tyrosine kinase inhibitors (TKIs) alone versus TKI + CNS-RT in terms of time to progression, iPFS or time to treatment failure [19].

In patients receiving radiation, larger metastases, neurological symptoms, and steroid use were more common. SRT is even suitable for BM treatment in oncogene-addicted NSCLC patients with four or more BMs [20]. With the increasing amounts of data that support concerns related to neurocognitive toxicity following WBRT, particularly in patients expected to have longer survival, our findings could also support the consideration of SRT + TKI or TKI alone in selected patients accompanied by close monitoring for intracranial progression to allow timely initiation of salvage treatment. This is similar to the results reported in the literature [21].

In the multivariate analysis, we confirmed that no smoking history, adenocarcinoma, lack of neurological symptoms, RPA class 1, and BMs with a maximum diameter Dmax ≤ 3 cm were associated with significantly better OS. The lack of neurological symptoms, RPA class 1, and BMs treated with targeted therapy were significant predictors of a better DBF-FS, which is consistent with other studies [14, 21]. An MRI radiomics method using clinical and radiomic features could predict the BM response to SRS as well as the treatment outcome, including local tumor control and survival [23]. Comprehensive genomic profiling is of clinical utility in assisting treatment selection, facilitating clinical trial enrollment, and improving patient outcomes in advanced NSCLC [24].

Neurological symptoms at the time of diagnosis of BM demonstrated a strong, independent prognostic impact on the survival prognosis of NSCLC [25]. Patients without neurological symptoms achieved obviously better OS and iPFS than patients with neurological symptoms in this study. We performed a subgroup analysis of BM patients with and without neurological symptoms. There were no significant differences in mOS, iPFS, or DBF-FS among the LINAC-SRS, WBRT and WBRT + RTB groups. However, in the subgroup analysis of treated patients without neurological symptoms, there were more patients treated with concurrent chemotherapy in the LINAC-SRS group than in the WBRT and WBRT + RTB groups. Patients with stable primary lesions were more likely to receive LINAC-SRS than WBRT or WBRT + RTB. Patients with stable primary disease had a longer survival period than those with progressive disease at the primary site, and SRS treatment could protect hippocampal function and improve the quality of life of patients, according to most research results. The median OS in the LINAC-SRS group and WBRT + RTB group had not yet been reached, and it was 39.6 months in the WBRT group.

Moreover, prophylactic cranial irradiation (PCI) gradually occurs during the course of NSCLC comprehensive treatment strategies. The PRoT-BM trial suggested that among a selected population with NSCLC with driver gene mutations who are at high risk for developing BMs, PCI significantly decreased the cumulative incidence of BMs in addition to increasing PFS and OS [26]. Another study suggested that PCI (25 Gy/10 fractions) showed limited benefit for survival outcomes and did not significantly decrease QoL or neurocognitive function, as measured using the Mini-Mental State Examination. We would continue to follow up these patients.

SRT or SRS is not yet available in some hospitals. Observation or delayed WBRT is an alternative approach. Hippocampal avoidance whole-brain radiotherapy might exert a protective effect on long-term neurocognitive function and did not affect patient survival. ASTRO clinical guidelines claim that for patients with a favorable prognosis and BMs receiving WBRT, hippocampal avoidance is recommended [27]. First-line TKIs plus concurrent cranial radiotherapy is a promising therapeutic strategy that leads to remarkable improvements in intracranial PFS and survival benefits for patients with EGFR-mutant NSCLC with BMs. A single-center retrospective study that included 184 EGFR/ALK-driven NSCLC patients with BMs found no independent effect due to first-line CNS treatment choice (WBRT, SRS or TKI) in EGFR/ALK-driven NSCLC, but the choice of first-line WBRT for BMs from EGFR/ALK-driven NSCLC was associated with a longer time to intracranial progression than SRS or TKI alone [21].

Within the European Organization for the Research and Treatment of Cancer (EORTC) 22952–26001 phase 3 trial, WBRT reduced the 2-year relapse rate both at initial sites and at new sites after radiosurgery or surgery in patients with 1–3 BMs and reduced neurological deaths, but it failed to improve the duration of functional independence and OS. SRS was associated with improved early local control of treated lesions compared with surgical resection, although the relative benefit decreased with time [28, 29].

The cumulative incidence of nervous toxicity in our study was 20.5% during our follow-up period, which is similar to other studies. But we did not found difference among three groups. The longer survival time maybe contribute this result. More studies supported the opinion that WBRT is associated with inferior quality of life and precognitive function. The incidence rates of memory decline in the groups of WBRT were significantly more increased than in the non-WBRT group [30]. In RTOG 0933 [31], a single-arm phase II study of WBRT without hippocampal avoidance (WBRT-HA), mean relative decline in Hopkins Verbal Learning Test–Revised Delayed Recall (HVLT-R DR) was magnificently lower(7.0% VS. 30%, 95% CI:4.7% to 18.7%, *P* < 0.001) in comparison with the historical control, a published phase III trial (PCI-P-120–9801). WBRT-HA might be another choose. In a word, the adverse reaction of nervous damage and outher toxicity attributed to intracranial RT is increased but tolerable with the prolongation of the survival time.

Recently, an increasing amount of data has indicated that immunotherapy (IT) combined with RT is better than RT alone for intracranial control and results in no increase in side effects. However, whether systemic treatment or PD-L1 expression affects the treatment effect is uncertain [10]. Intracranial progression in patients treated with PD-l/PD-L1 inhibitors predominantly occurred at the original BM sites. The use of upfront cranial radiotherapy may improve OS, especially in NSCLC patients with 1–4 BMs [32]. Nevertheless, the intervention time of IT and RT remains controversial. Appropriate IT-SRS combinations have been envisioned as a strategy for the management of patients with BMs.

There are some limitations to the present study. First, this is a retrospective study with patient selection bias. Second, some variables, such as gene detection details, were not evaluated in the study. Third, the development of RT and targeted therapy tended to lead to bias, as patients from 2010 to 2019 were enrolled in this study. Fourth, the data were be extracted from the electronic medical record. Despite these limitations, this real-world study provides certain evidence for individual clinical treatment decisions and can inform prognostic considerations in the management of NSCLC patients with 1–4 BMs.

## Conclusion

In conclusion, LINAC-SRS did not result in significantly superior survival, intracranial control or neurocognitive function protection compared to WBRT or WBRT plus boost when used to treat patients with NSCLC with 1–4 BMs. RPA class and neurological symptoms were independent factors associated with worse OS and DBF-FS. The use of targeted therapy after the detection of BMs is an independent factor associated with iPFS and DBF-FS. Additional studies are warranted to confirm these findings. The identification of methods to protect the hippocampus and reduce adverse reactions to the nervous system while performing intracranial radiotherapy should be the focus of future research.

## Supplementary Information

**Additional file 1. **Trial profile 

**Additional file 2. **Univariate and multivariate analysis of DBF-FS.

**Additional file 3. **Baseline characteristics among the three groups of patients with neurological symptoms. 

**Additional file 4. **Baseline characteristics among the three groups of patients without neurological symptoms. 

## Data Availability

The datasets generated during and/or analyzed during the current study are available from the corresponding author on reasonable request.

## Abbreviations

BMs: Brain metastases
CI: Confidence interval
DBF-FS: Distant brain failure-free survival
EMs: Extracranial metastases
GPA: Graded prognostic assessment
GTV: Gross tumor volume
HR: Hazard ratio
iPFS: Intracranial progression-free survival
iRT: Intracranial radiotherapy
LINAC-SRS: Stereotactic radiotherapy using linear accelerators
NSCLC: Non-small cell lung cancer
OS: Overall survival
KPS: Kanofsky Performance Scale
PTV: Planning target volume
RPA: Recursive partitioning analysis
RT: Radiotherapy
TT: Targeted therapy
WBRT: Whole-brain radiotherapy
WBRT + RTB: Whole-brain radiotherapy plus radiotherapy boost
WBRT-HA: Hippocampus-avoidance whole-brain radiotherapy
