# Supplementary figures and images for: Real-world analysis of different intracranial radiation therapies in non-small cell lung cancer patients with 1–4 brain metastases
Source: BMC Cancer. 2022 Sep 24;22:1010. doi: 10.1186/s12885-022-10083-8 (PMC9508739; doi:10.1186/s12885-022-10083-8)

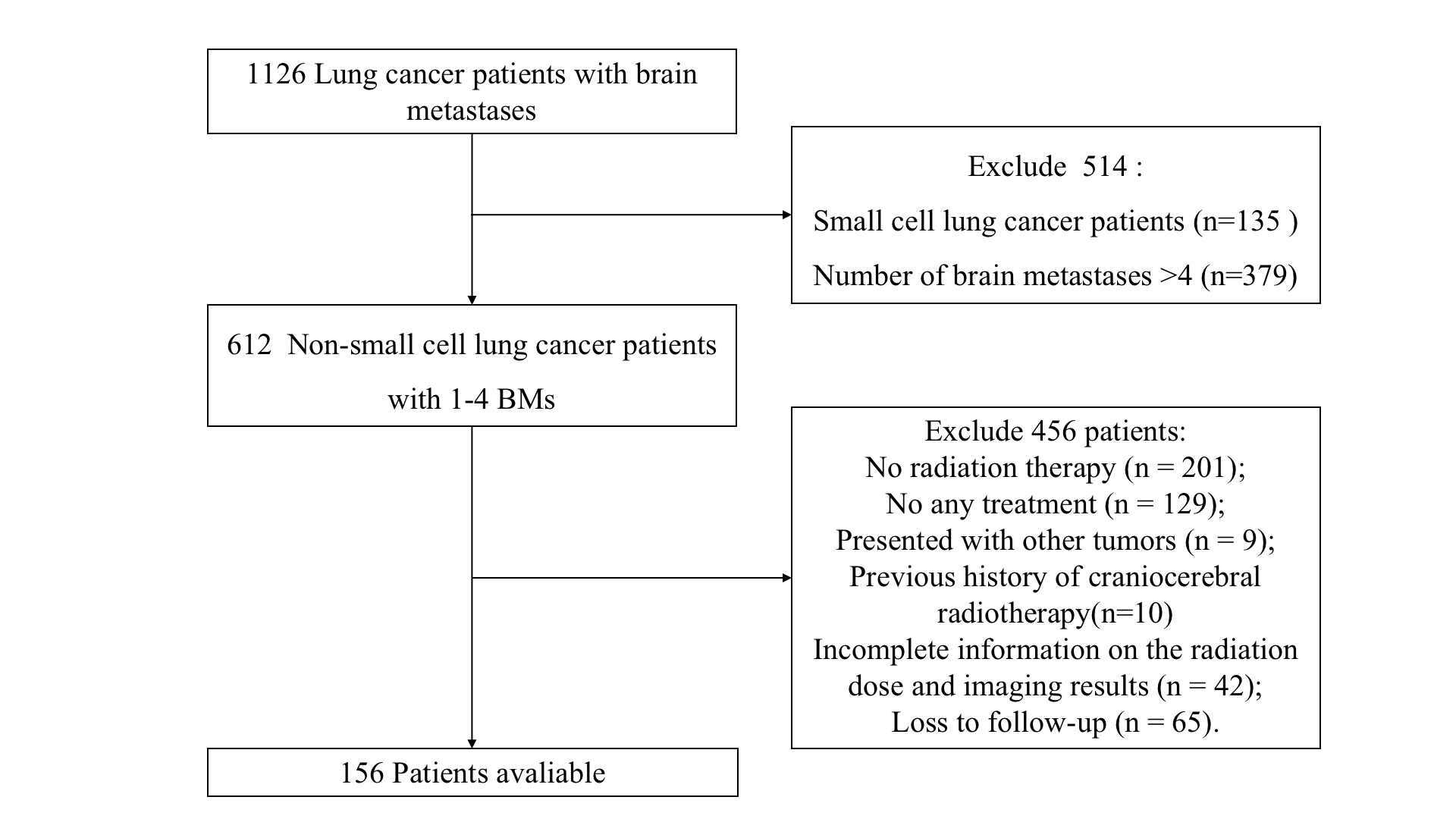

Supplement: Supplementary file 1 — Additional file 1. Trial profile [file 12885_2022_10083_MOESM1_ESM.tif]
