# Supplementary material for: Real-world analysis of different intracranial radiation therapies in non-small cell lung cancer patients with 1–4 brain metastases
Source: BMC Cancer. 2022 Sep 24;22:1010. doi: 10.1186/s12885-022-10083-8 (PMC9508739; doi:10.1186/s12885-022-10083-8)
Supplement: Supplementary file 2 — Additional file 2. Univariate and multivariate analysis of DBF-FS. [file 12885_2022_10083_MOESM2_ESM.docx]

Additional file 2.1 Univariate analysis of DBF-FS

| Parameter | DBF-FS(m) | *P* |  | Parameter | DBF-FS(m) | *P* |
| --- | --- | --- | --- | --- | --- | --- |
| Sex |  | 0.515 |  | Neurologic symptoms |  | 0.002 |
| Female | 27.1 |  |  | No | 27.3 |  |
| Male | 19.2 |  |  | Yes | 15.0 |  |
| Age, years |  | 0.896 |  | Number of BMs |  | 0.450 |
| ≤50 | 16.2 |  |  | 1 | 24.0 |  |
| ≥51 | 19.4 |  |  | 2-4 | 17.9 |  |
| Smoking status |  | 0.157 |  | BM size, Dmax(cm) |  | 0.091 |
| Never smoker | 27.1 |  |  | ≤3 | 21.1 |  |
| Current/ex-smoker | 18.2 |  |  | >3 | 16.2 |  |
| KPS scores |  | 0.459 |  | Primary disease control |  | 0.597 |
| ≥90 | 19.5 |  |  | Yes | 19.4 |  |
| ≤80 | 18.4 |  |  | No | 18.4 |  |
| Tumor histology |  | 0.255 |  | EMs |  | 0.203 |
| SCC | 19.4 |  |  | No | 21.1 |  |
| Adenocarcinoma | 19.2 |  |  | Yes | 16.6 |  |
| Thoracic operation |  | 0.141 |  | RPA class |  | 0.009 |
| Yes | 27.1 |  |  | 1 | 33.2 |  |
| No | 18.2 |  |  | 2 | 16.6 |  |
| Initial treatment of BMs |  | 0.796 |  | Concurrent chemotherapy |  | 0.302 |
| Yes | 21.3 |  |  | Yes | 15.2 |  |
| No | 19.2 |  |  | No | 21.1 |  |
| GPA scores |  | 0.727 |  | TT after BMs |  | 0.046 |
| 0.5-1.5 | 21.1 |  |  | Yes | 27.9 |  |
| 2-2.5 | 18.2 |  |  | No | 16.2 |  |
| ≥3 | 26.4 |  |  |  |  |  |

Additional file 2.2 Multivariate analysis of DBF-FS

| Parameter | DBF-FS (m) | HR | 95%CI | *P* |
| --- | --- | --- | --- | --- |
| Neurologic symptoms |  |  |  | 0.007 |
| No | 27.3 | 1.000 |  |  |
| Yes | 15.0 | 1.874 | 1.189-2.954 |  |
| BM size, Dmax(cm) |  |  |  | 0.528 |
| ≤3 | 21.1 | 1.000 |  |  |
| >3 | 16.2 | 1.203 | 0.678-2.136 |  |
| RPA class |  |  |  | 0.021 |
| 1 | 33.2 | 1.000 |  |  |
| 2 | 16.6 | 1.812 | 1.094-3.000 |  |
| TT after BMs |  |  |  | 0.011 |
| Yes | 27.9 | 1.000 |  |  |
| No | 16.2 | 1.932 | 1.164-3.206 |  |

BMs, brain metastases; CI, confidence interval; DBF-FS, distant brain failure-free survival;

EMs, extracranial metastases; GPA, graded prognostic assessment; HR, hazard ratio; KPS, Karnofsky Performance Scale; RPA, recursive partitioning analysis; RTB, radiotherapy boost;

TT, targeted therapy; WBRT, whole-brain radiotherapy;
