# Supplementary material for: Real-world analysis of different intracranial radiation therapies in non-small cell lung cancer patients with 1–4 brain metastases
Source: BMC Cancer. 2022 Sep 24;22:1010. doi: 10.1186/s12885-022-10083-8 (PMC9508739; doi:10.1186/s12885-022-10083-8)
Supplement: Supplementary file 3 — Additional file 3. Baseline characteristics among the three groups of patients with neurological symptoms. [file 12885_2022_10083_MOESM3_ESM.docx]

Additional file 3. Baseline characteristics among the three groups of patients with neurologic symptoms.

| Characteristics | All  N（%） | LINAC-SRS  N（%） | WBRT  N（%） | WBRT + RTB N（%） | P |
| --- | --- | --- | --- | --- | --- |
| Number of patients | 69 (100) | 32(46.4) | 19(27.5) | 18(26.1) |  |
| Sex |  |  |  |  | 0.150 |
| Female | 27(39.1) | 16(50.0) | 7(36.8) | 4(22.2) |  |
| Male | 42(60.9) | 16(50.0) | 12(63.2) | 14(77.8) |  |
| Age, years |  |  |  |  | 0.169 |
| ≤50 | 21(30.4) | 13(40.6) | 3(15.8) | 5(27.8) |  |
| ≥51 | 48 (69.9) | 19(59.4) | 16(84.2) | 13(72.2) |  |
| Smoking status |  |  |  |  | 0.180 |
| Never smoker | 35(50.7) | 17(53.1) | 12(63.2) | 6(33.3) |  |
| Current/ex-smoker | 34(49.3) | 15(46.9) | 7(36.8) | 12(66.7) |  |
| KPS scores |  |  |  |  | 0.332 |
| ≥90 | 43(62.3) | 17(53.1) | 13(68.4) | 13 (72.2) |  |
| ≤80 | 26(37.7) | 15(46.9) | 6(31.6) | 5(27.8) |  |
| Tumor histology |  |  |  |  | 0.375 |
| Squamous cell carcinoma | 11(15.9) | 7(21.9) | 3(15.8) | 1(5.6) |  |
| Adenocarcinoma | 58(84.1) | 25(78.1) | 16(84.2) | 17(94.4) |  |
| Thoracic operation |  |  |  |  | 0.657 |
| Yes | 21 (30.4) | 9(28.1) | 5(26.3) | 7(38.9) |  |
| No | 48(69.6) | 32(71.9) | 14(73.7) | 11(61.1) |  |
| Initial treatment of BMs |  |  |  |  | 0.420 |
| Yes | 36 (52.2) | 18(56.2) | 11(57.9) | 7(38.9) |  |
| No | 33(47.8) | 14(43.8) | 8(42.1) | 11(61.1) |  |
| Number of BMs |  |  |  |  | 0.765 |
| 1 | 40(58.0) | 20(62.5) | 10 (52.6) | 10(55.6) |  |
| 2-4 | 29 (42.0) | 12(37.5) | 9(47.4) | 8(44.4) |  |
| BM size, Dmax(cm) |  |  |  |  | 0.038 |
| ≤3 | 48(69.6) | 26(81.3) | 9(47.4) | 13 (72.2) |  |
| >3 | 21(30.4) | 6(18.7) | 10(52.6) | 5(27.8) |  |
| Primary disease control |  |  |  |  | 0.213 |
| Yes | 55(79.7) | 28(87.5) | 15(78.9) | 12 (66.7) |  |
| No | 14(20.3) | 4(12.5) | 4(21.1) | 6(33.3) |  |
| EMs |  |  |  |  | 0.462 |
| Yes | 42 (60.9) | 19(59.4) | 10 (52.6) | 13(72.2) |  |
| No | 27(39.1) | 13(40.6) | 9(47.4) | 5(27.8) |  |
| RPA class |  |  |  |  | 0.149 |
| 1 | 15 (21.7) | 9 (28.1) | 5(26.3) | 1(5.6) |  |
| 2 | 54 (78.3) | 23(71.9) | 14(73.7) | 17(94.4) |  |
| GPA scores |  |  |  |  | 0.787 |
| 0.5-1.5 | 12(17.4) | 4 (12.5) | 4(21.0) | 4(22.2) |  |
| 2-2.5 | 29(42.0) | 13(40.6) | 9(47.4) | 7(38.9) |  |
| ≥3 | 28(40.6) | 15(46.9) | 6(31.6) | 7(38.9) |  |
| Concurrent chemotherapy |  |  |  |  | 0.920 |
| Yes | 23(33.3) | 10 (31.2) | 7(36.8) | 6(33.3) |  |
| No | 46(66.7) | 22(68.8) | 12(63.2) | 12(67.7) |  |
| TT after BMs |  |  |  |  | 0.841 |
| Yes | 24(34.8) | 10(31.2) | 7(36.8) | 7(38.9) |  |
| No | 45(65.2) | 22(68.8) | 12(63.2) | 11(61.1) |  |

Abbreviations: BMs, brain metastases;EMs, extracranial metastases; GPA, graded prognostic assessment; HR, hazard ratio; KPS, Karnofsky Performance Scale; RPA, recursive partitioning analysis; RTB, radiotherapy boost;TT, targeted therapy; WBRT, whole-brain radiotherapy.
