# Supplementary material for: Real-world analysis of different intracranial radiation therapies in non-small cell lung cancer patients with 1–4 brain metastases
Source: BMC Cancer. 2022 Sep 24;22:1010. doi: 10.1186/s12885-022-10083-8 (PMC9508739; doi:10.1186/s12885-022-10083-8)
Supplement: Supplementary file 4 — Additional file 4. Baseline characteristics among the three groups of patients without neurological symptoms. [file 12885_2022_10083_MOESM4_ESM.docx]

Additional file 4. Baseline characteristics among the three groups of patients without neurologic symptoms.

| Characteristics | All  N（%） | LINAC-SRS  N（%） | WBRT  N（%） | WBRT + RTB N（%） | P |
| --- | --- | --- | --- | --- | --- |
| Number of patients | 87 (100) | 45(51.7) | 25(28.8) | 17(19.5) |  |
| Sex |  |  |  |  | 0.612 |
| Female | 31(35.6) | 17(37.8) | 7(28.0) | 7(41.2) |  |
| Male | 56(64.4) | 28(62.2) | 18(72.0) | 10(58.8) |  |
| Age, years |  |  |  |  | 0.435 |
| ≤50 | 36(41.4) | 17(37.8) | 13(52.0) | 6(35.3) |  |
| ≥51 | 51 (58.6) | 28(62.2) | 12(48.0) | 11(64.7) |  |
| Smoking status |  |  |  |  | 0.176 |
| Never smoker | 41(47.1) | 17(37.8) | 15(60.0) | 9(52.9) |  |
| Current/ex-smoker | 46(52.9) | 28(62.2) | 10(40.0) | 8(47.1) |  |
| KPS scores |  |  |  |  | 0.705 |
| ≥90 | 34(39.1) | 16(35.6) | 10(40.0) | 8 (47.1) |  |
| ≤80 | 53(60.9) | 29(64.4) | 15(60.0) | 9(52.9) |  |
| Tumor histology |  |  |  |  | 0.117 |
| Squamous cell carcinoma | 11(12.6) | 9(20.0) | 1(4.0) | 1(5.9) |  |
| Adenocarcinoma | 76(87.4) | 36(80.0) | 24(96.0) | 16(94.1) |  |
| Thoracic operation |  |  |  |  | 0.839 |
| Yes | 47(54.0) | 23(51.1) | 14(56.0) | 10(58.8) |  |
| No | 40(46.0) | 22(48.9) | 11(44.0) | 7(41.2) |  |
| Initial treatment of BM^a^ |  |  |  |  | 0.057 |
| Yes | 30 (34.5) | 12(26.7) | 8(32.0) | 10(58.8) |  |
| No | 57(65.5) | 33(73.3) | 17(68.0) | 7(41.2) |  |
| Number of BMs |  |  |  |  | 0.536 |
| 1 | 51(58.6) | 25(55.6) | 14 (56.0) | 12(70.6) |  |
| 2-4 | 36 (41.4) | 20(44.4) | 11(44.0) | 5(29.4) |  |
| BM size, Dmax(cm) |  |  |  |  | 1.000 |
| ≤3 | 82(94.3) | 42(93.3) | 24(96.0) | 16 (94.1) |  |
| >3 | 5(5.7) | 3(6.7) | 1(4.0) | 1(5.9) |  |
| Primary disease control |  |  |  |  | 0.001 |
| Yes | 70(80.5) | 42(93.3) | 14(56.0) | 14 (82.4) |  |
| No | 17(19.5) | 3(6.7) | 11(44.0) | 3(17.6) |  |
| EMs |  |  |  |  | 0.338 |
| Yes | 34 (39.1) | 19(42.2) | 11(44.0) | 4(23.5) |  |
| No | 53(60.9) | 26(57.8) | 14(56.0) | 13(76.5) |  |
| RPA class |  |  |  |  | 0.301 |
| 1 | 37 (42.5) | 18 (40.0) | 9(36.0) | 10(58.8) |  |
| 2 | 50 (57.5) | 27(60.0) | 16(64.0) | 7(41.2) |  |
| GPA scores |  |  |  |  | 0.311 |
| 0.5-1.5 | 10(11.5) | 4 (8.9) | 4(16.0) | 2(11.8) |  |
| 2-2.5 | 33(37.9) | 20(44.4) | 10(40.0) | 3(17.6) |  |
| ≥3 | 44(50.6) | 21(46.7) | 11(44.0) | 12(70.6) |  |
| Concurrent chemotherapy |  |  |  |  | 0.023 |
| Yes | 19(21.8) | 15 (33.3) | 3(12.0) | 1(5.9) |  |
| No | 68(78.2) | 30(66.7) | 22(88.0) | 16(94.1) |  |
| TT after BMs |  |  |  |  | 0.055 |
| Yes | 23(26.4) | 7(15.6) | 9(36.0) | 7(41.2) |  |
| No | 64(73.6) | 38(84.4) | 16(64.0) | 10(58.8) |  |

Abbreviations: BMs, brain metastases;EMs, extracranial metastases; GPA, graded prognostic assessment; KPS, Karnofsky Performance Scale; RPA, recursive partitioning analysis; RTB, radiotherapy boost;TT, targeted therapy; WBRT, whole-brain radiotherapy.^a^ NSCLC patients have BMs at diagnosis.
